# Protein Kinase A Governs Oxidative Phosphorylation Kinetics and Oxidant Emitting Potential at Complex I

**DOI:** 10.3389/fphys.2015.00332

**Published:** 2015-11-17

**Authors:** Daniel S. Lark, Lauren R. Reese, Terence E. Ryan, Maria J. Torres, Cody D. Smith, Chien-Te Lin, P. Darrell Neufer

**Affiliations:** ^1^East Carolina Diabetes and Obesity InstituteGreenville, NC, USA; ^2^Department of Kinesiology, East Carolina UniversityGreenville, NC, USA; ^3^Department of Physiology, Brody School of Medicine, East Carolina UniversityGreenville, NC, USA

**Keywords:** mitochondria, adenylyl cyclase, cAMP, protein kinase A, complex I, respiration, skeletal muscle, liver

## Abstract

The mitochondrial electron transport system (ETS) is responsible for setting and maintaining both the energy and redox charges throughout the cell. Reversible phosphorylation of mitochondrial proteins, particularly via the soluble adenylyl cyclase (sAC)/cyclic AMP (cAMP)/Protein kinase A (PKA) axis, has recently been revealed as a potential mechanism regulating the ETS. However, the governance of cAMP/PKA signaling and its implications on ETS function are incompletely understood. In contrast to prior reports using exogenous bicarbonate, we provide evidence that endogenous CO_2_ produced by increased tricarboxylic acid (TCA) cycle flux is insufficient to increase mitochondrial cAMP levels, and that exogenous addition of membrane permeant 8Br-cAMP does not enhance mitochondrial respiratory capacity. We also report important non-specific effects of commonly used inhibitors of sAC which preclude their use in studies of mitochondrial function. In isolated liver mitochondria, inhibition of PKA reduced complex I-, but not complex II-supported respiratory capacity. In permeabilized myofibers, inhibition of PKA lowered both the K_*m*_ and V_*max*_ for complex I-supported respiration as well as succinate-supported H_2_O_2_ emitting potential. In summary, the data provided here improve our understanding of how mitochondrial cAMP production is regulated, illustrate a need for better tools to examine the impact of sAC activity on mitochondrial biology, and suggest that cAMP/PKA signaling contributes to the governance of electron flow through complex I of the ETS.

## Introduction

Mitochondrial function is a key determinant of skeletal muscle metabolic health since it governs both the energetic and redox environments of the myocyte. Under conditions of overnutrition and/or obesity, evidence suggests mitochondria from humans and rodents increase their rate of hydrogen peroxide (H_2_O_2_) emission (Houstis et al., 2006; Fisher-Wellman et al., 2014), with the resulting increase in oxidative burden impairing skeletal muscle insulin action (Anderson et al., 2009; Hoehn et al., 2009; Lark et al., 2015). Key enzymes within the electron transport system and the matrix regulate the rate of H_2_O_2_ production and scavenging that ultimately determine the rate of H_2_O_2_ release. Therefore, a better understanding of how mitochondrial enzymes are regulated may lead to better treatments for diseases like diabetes that are linked to mitochondrial H_2_O_2_ emission.

Mounting evidence implicates post-translational modifications to mitochondrial proteins, particularly phosphorylation events mediated by the cyclic adenosine monophosphate (cAMP)/Protein kinase A (PKA) axis, as a key regulator of cellular metabolism (Valsecchi et al., 2013; Di Benedetto et al., 2014). Mitochondrial cAMP/PKA signaling is thought to be initiated by soluble adenylyl cyclase (sAC) (Buck et al., 1999), a bicarbonate (${\text{HCO}}_{3}^{-}$)- and Ca^2+^-activated (Chen et al., 2000) enzyme that generates cAMP in various intracellular compartments (e.g., mitochondrial matrix) (Zippin et al., 2003). The implication is that CO_2_ generated during accelerated flux through the tricarboxylic acid (TCA) cycle is converted to ${\text{HCO}}_{3}^{-}$ via carbonic anhydrase (CA) and activates the mitochondrial cAMP/PKA axis. However, although it is well-established that exogenous ${\text{HCO}}_{3}^{-}$ can activate mitochondrial sAC (Chen et al., 2000; Zippin et al., 2003), it is not known whether increased endogenous metabolic CO_2_ production increases mitochondrial cAMP.

Analysis of the MitoCarta mitochondrial proteome database (Pagliarini et al., 2008) has revealed approximately 75 different putative targets of PKA-mediated phosphorylation, some of which are altered by dietary manipulation (Grimsrud et al., 2012). Available evidence suggests cAMP/PKA signaling alters oxidative phosphorylation (OXPHOS) by regulating cytochrome C oxidase (Acin-Perez et al., 2009a,b, 2010) or enhancing ATP production in the presence of Ca^2+^ (Di Benedetto et al., 2013). Additionally, several independent groups have identified Complex I of the electron transport system (ETS) as a target of PKA-dependent phosphorylation (Papa, 2002; De Rasmo et al., 2010) with a potential role in a number of human pathologies (Valenti et al., 2011; Papa et al., 2012). Despite the cummulative evidence implicating cAMP/PKA-mediated regulation of the ETS in human disease, the potential functional impact of cAMP/PKA-mediated phosphorylation on mitochondrial bioenergetics is not well understood.

Therefore, the purpose of the present study was to determine: (1) if endogenous CO_2_ production from the TCA cycle is sufficient to increase mitochondrial cAMP levels and (2) whether PKA acts on multiple ETS complexes (including Complex I) as a feed-forward mechanism to enhance OXPHOS in response to metabolic demand.

## Methods

### Chemicals and reagents

All chemicals and reagents were obtained from Sigma Aldrich except for Amplex Ultra Red reagent, which was purchased from Molecular Probes Inc.

### Animal use procedures

All aspects of rodent studies were approved by the East Carolina University Animal Care and Use Committee. Male C57BL6/NJ mice were purchased from Jackson Laboratories and were the only model used in these studies. Mice were housed in a temperature- (22°C) and light-controlled room and given free access to food and water. At the time of experiment, mice were 8–12 weeks of age.

### Mitochondrial isolation

For mitochondrial isolation, mice were anesthetized by inhalation of isoflurane following a 4 h fast and were euthanized via double pneumothorax. Under anesthesia, liver, or hind limb muscles (gastrocnemius, quadriceps, and biceps femoris) were immediately excised and rinsed in ice-cold mitochondrial isolation medium (MIM) containing: 300 mM Sucrose, 10 mM HEPES, and 1 mM EGTA. Tissues were then transferred to a dry dish and minced continuously for 5 min, then transferred to a 50 ml tube containing 10 ml of MIM. For skeletal muscle, trypsin (100 mg/ml) was added for exactly 2 min, then soybean trypsin inhibitor in 10 ml of MIM + 1 mg/ml BSA was added to halt the reaction. Tissue was then gently mixed by inversion and allowed to settle to the bottom of the tube. Supernatant was discarded and tissue re-suspended in MIM+BSA (20 ml/g tissue). Minced liver was not treated with trypsin. Tissues were then homogenized using a tight-fitting Teflon glass homogenizer (~10 passes) and centrifuged at 800 g for 10 min at 4°C. Supernatant was transferred to Oakridge tubes and centrifuged at 8000 g for 15 min at 4°C. Supernatant was discarded and pellet was washed and re-suspended in 10 ml of MIM+BSA and centrifuged again at 8000 g for 15 min at 4°C. The final pellet was re-suspended in 50 μl of MIM. Mitochondrial protein concentration was determined by spectrophotometry using the bicinchoninic acid method (Pierce). In some experiments, mitochondria were fractured by three freeze-thaw cycles and directly assayed for complex I specific activity (Barrientos et al., 2009).

### Preparation of mouse permeabilized myofiber bundles (PmFBs)

The PmFB technique used was partially adapted from previous methods (Kuznetsov et al., 1996; Tonkonogi et al., 2003) and has been described previously (Anderson and Neufer, 2006). Mice were anesthetized by inhalation of isoflurane and the red (RG) and white (WG) portions of the gastrocnemius muscle were immediately excised. Muscle samples were placed in ice-cold (4°C) Buffer X containing (in mM): 7.23 K_2_EGTA, 2.77 CaK_2_EGTA, 20 Imidazole, 20 Taurine, 5.7 ATP, 14.3 Phosphocreatine, 6.56 MgCl_2_-6H_2_O, and 50 MES (pH 7.1, 295 mOsm). Under a dissecting microscope (Leica Optics), fat, and connective tissue were removed and muscle samples were separated into small bundles of fibers (< 1 mg wet weight/fiber bundle). Fiber bundles were permeabilized in Buffer X supplemented with 40 μg/ml saponin, a mild, cholesterol-specific detergent for 30 min at 4°C as previously described (Anderson and Neufer, 2006). Since the sarcolemmal membrane contains a large amount of cholesterol relative to the mitochondrial membrane, this technique selectively permeabilizes the sarcolemma while leaving mitochondrial membranes and ultra-structure intact (Kuznetsov et al., 2008; Picard et al., 2011). PmFBs were then washed in ice-cold Buffer Z containing (in mM): 105 K-MES, 30 KCl, 5 KH_2_PO_4_, 5 MgCl_2_-6H_2_O, and 0.5 mg/ml Bovine serum albumin (pH 7.1, 295 mOsm) and remained in Buffer Z on a rotator at 4°C until analysis (< 4 h).

### Mitochondrial camp production assay

Two different sets of experiments were done to measure mitochondrial cAMP production in the current study. First, isolated liver mitochondria were incubated for 10 min at 37°C in 300 μl of MAITE medium containing (in mM): 10 Tris-HCl, 25 sucrose, 75 sorbitol, 100 KCl, 0.5 EDTA, 5 MgCl_2_, and 1 mg/ml BSA; pH 7.4. MAITE medium was also supplemented with 300 mM HEPES to maintain pH in the presence of ${\text{HCO}}_{3}^{-}$ (Acin-Perez et al., 2009b) and 1 mM ATP as substrate for cAMP production. Experiments were performed under three conditions: no additions, 30 mM ${\text{HCO}}_{3}^{-}$ and ${\text{HCO}}_{3}^{-}$ plus 25 μM KH7, an inhibitor of sAC (Hess et al., 2005). Second, skeletal muscle mitochondria (250 μg/ml) were incubated at 37°C in 300 μl of MAITE medium supplemented with 1 mM ATP, 10 μg/ml oligomycin and in the presence or absence of 25 μM KH7 or 5 μM acetazolamide (AZA), a carbonic anhydrase inhibitor to prevent conversion of CO_2_ to ${\text{HCO}}_{3}^{-}$ (Maren, 1960). Following an initial 10 min acclimation period, mitochondria were incubated for 5 min in the presence of the following respiratory substrate combinations: 5 pyruvate/2 mM malate, 5 mM succinate, or 25 μM palmitoyl-L-carnitine/2 mM malate. A separate set of control samples did not receive respiratory substrates. In some experiments, 1 μM FCCP was added to uncouple O_2_ consumption from ATP synthesis and thereby accelerate TCA cycle flux. Reactions were halted by the addition of 0.1 M HCl, and then samples were flash frozen and stored in liquid N_2_ until analysis for cAMP (Complete cAMP ELISA Kit, Enzo Life Sciences).

### Mitochondrial bioenergetics assays

Mitochondrial respiration experiments in both isolated mitochondria and PmFBs were performed using a high-resolution oxygraph (Oroboros O_2_k, Innsbruck Austria). Respirometry experiments using isolated mitochondria were performed in Buffer Z at 25°C while substrate titration experiments in PmFBs were performed at 37°C in Buffer Z supplemented with 20 mM creatine monohydrate to maximize phosphate transfer in PmFBs (Kuznetsov et al., 1996). Blebbistatin (20 μl) was also added during PmFB experiments to mitigate the effects of contraction on respiratory kinetics (Perry et al., 2011).

Mitochondrial H_2_O_2_ emitting potential, defined as the H_2_O_2_ that escapes the matrix, was measured via Amplex Ultra Red (Invitrogen) fluorescence detected at 565/600 ex/em at 37°C in a monochromatic spectrofluorometer (Horiba Jobin-Yvon) with Buffer Z as previously described (Anderson and Neufer, 2006). Assays were performed in the presence of 25 U/ml superoxide dismutase to ensure superoxide produced and released on the outer surface of the mitochondrial inner membrane was converted to H_2_O_2_. Mitochondrial H_2_O_2_ emitting potential in PmFBs was measured during either reverse electron flow using 5 mM succinate or forward electron flow using 5 mM glutamate and 2 mM malate followed by the addition of rotenone (Lambert and Brand, 2004). Once steady-state rates of H_2_O_2_ emission were established (< 10 min), 1 μM auranofin, a thioredoxin reductase inhibitor, was added to remove oxidant scavenging as a potentially confounding factor (Fisher-Wellman et al., 2013). In addition to yielding a measure of H_2_O_2_ production, this allowed for the determination of oxidant scavenging capacity as the difference in H_2_O_2_ emission before and after the addition of auranofin.

In experiments utilizing 8Br-cAMP or H89, compounds were added to the oxygraph chamber or cuvette with isolated mitochondria or PmFBs for 10 min prior to any subsequent additions.

### Statistical analyses

Comparisons between control and treatment groups were made using One-way ANOVA with Student Newman-Keuls *post-hoc* test where appropriate using Prism statistical software (GraphPad Prism 6). Pair-wise comparisons were made using student's paired two-way *t*-test. In all experiments, data are reported as mean ± SEM unless otherwise noted. Significance level was set at *p* < 0.05.

## Results

### TCA cycle flux does not increase [cAMP] in isolated mitochondria

**Figure 1:**
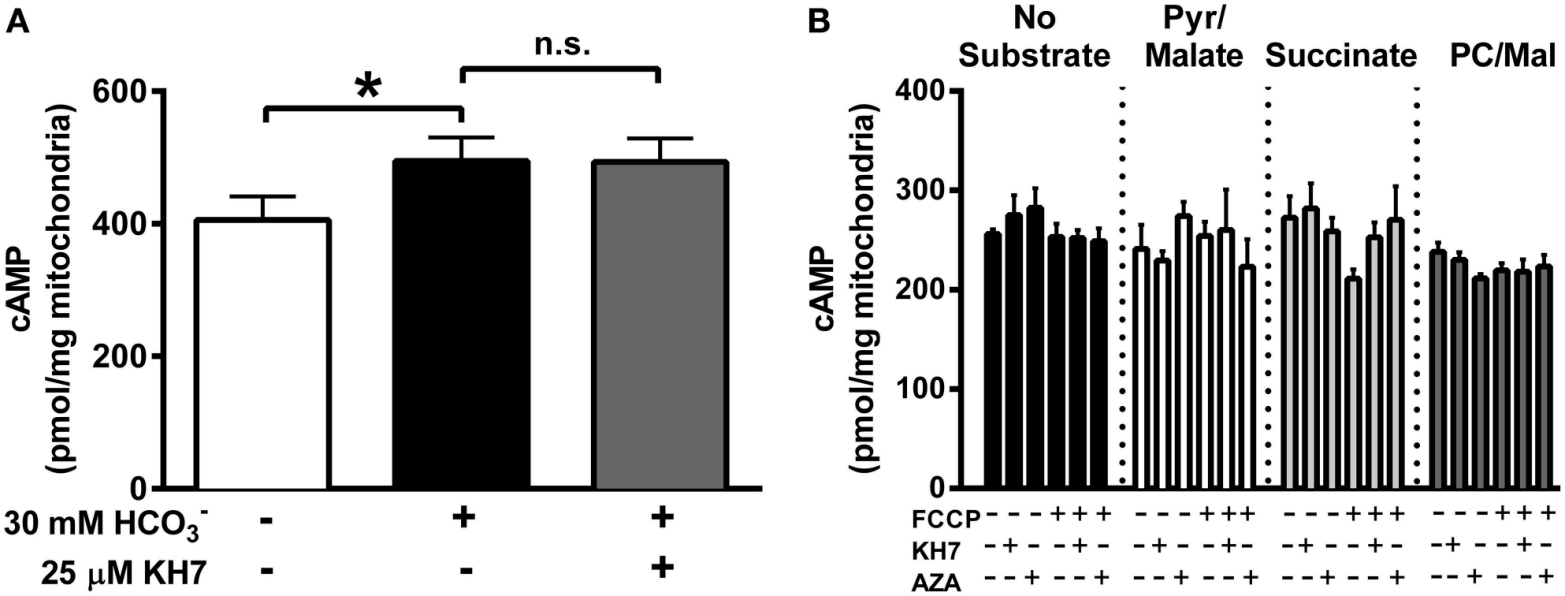
**TCA-cycle dependent flux does not increase mitochondrial cAMP**. **(A)** cAMP was measured in isolated liver mitochondria in the presence of 1 mM ATP, and in the absence of inhibitors (white bar), the presence of 30 mM ${\text{HCO}}_{3}^{-}$ (black bar) or the presence of both ${\text{HCO}}_{3}^{-}$ and 25 μM KH7 (gray bar). *N* = 4/condition. * denotes *p* < 0.05 compared to untreated condition. **(B)** cAMP was measured in isolated skeletal muscle mitochondria in the presence of 1 mM ATP, and in the absence (black bars) of respiratory substrates or in the presence of pyruvate/malate (white bars), succinate (light gray bars) or palmitoyl-L-carnitine/malate (dark gray bars). For each round of experiments, a single mitochondrial preparation was used for all substrate conditions, including parallel experiments with FCCP, KH7, and acetazolamide (AZA). *N* = 4 mitochondrial preparations from individual mice.

The inner mitochondrial membrane is impermeable to cytosolic cAMP (Di Benedetto et al., 2013) and, therefore, matrix cAMP has been proposed to be generated locally by CO_2_-mediated activation of sAC. Evidence in support of this hypothesis comes from data showing that addition of exogenous ${\text{HCO}}_{3}^{-}$ induces a small increase (~10%) in mitochondrial cAMP that is prevented by the sAC inhibitor KH7 (Chen et al., 2000; Litvin et al., 2003; Zippin et al., 2003; Acin-Perez et al., 2009b; Di Benedetto et al., 2013). In the present study, addition of ${\text{HCO}}_{3}^{-}$ to isolated liver mitochondria generated a small but significant increase in cAMP that, in contrast to previous findings (Acin-Perez et al., 2009b), was not blunted by KH7 (Figure 1A).

The TCA cycle has been proposed as the source of CO_2_ needed to activate sAC in mitochondria (Acin-Perez et al., 2009b), although this has yet to be demonstrated experimentally. Using isolated mitochondria from skeletal muscle in the presence of 1 mM ATP, we were unable to detect any increase in cAMP during respiration supported by CO_2_-generating substrates (pyruvate/malate or palmitoyl-L-carnitine/malate) compared with mitochondria in the absence of substrate or those oxidizing non CO_2_-generating substrates (succinate) (Figure 1B). Mitochondrial cAMP remained unchanged even when TCA cycle flux was accelerated by the mitochondrial uncoupler FCCP. Finally, consistent with the data from liver mitochondria (Figure 1A), cAMP levels were not decreased by the putative sAC inhibitor KH7 or acetazolamide (AZA), a carbonic anhydrase inhibitor. These findings suggest that, at least under the conditions tested, endogenous TCA cycle-derived CO_2_ production is not sufficient to increase mitochondrial cAMP in skeletal muscle mitochondria.

### Regulation of OXPHOs function by mitochondrial cAMP/PKA signaling

The functional consequence of mitochondrial cAMP/PKA signaling on OXPHOS function is unclear as exogenous activation of PKA has been reported to either increase (Acin-Perez et al., 2009b, 2011) or decrease (Di Benedetto et al., 2013) mitochondrial ATP production. Here, a series of experiments were performed to test the hypothesis that OXPHOS is regulated by mitochondrial cAMP/PKA signaling.

**Figure 2:**
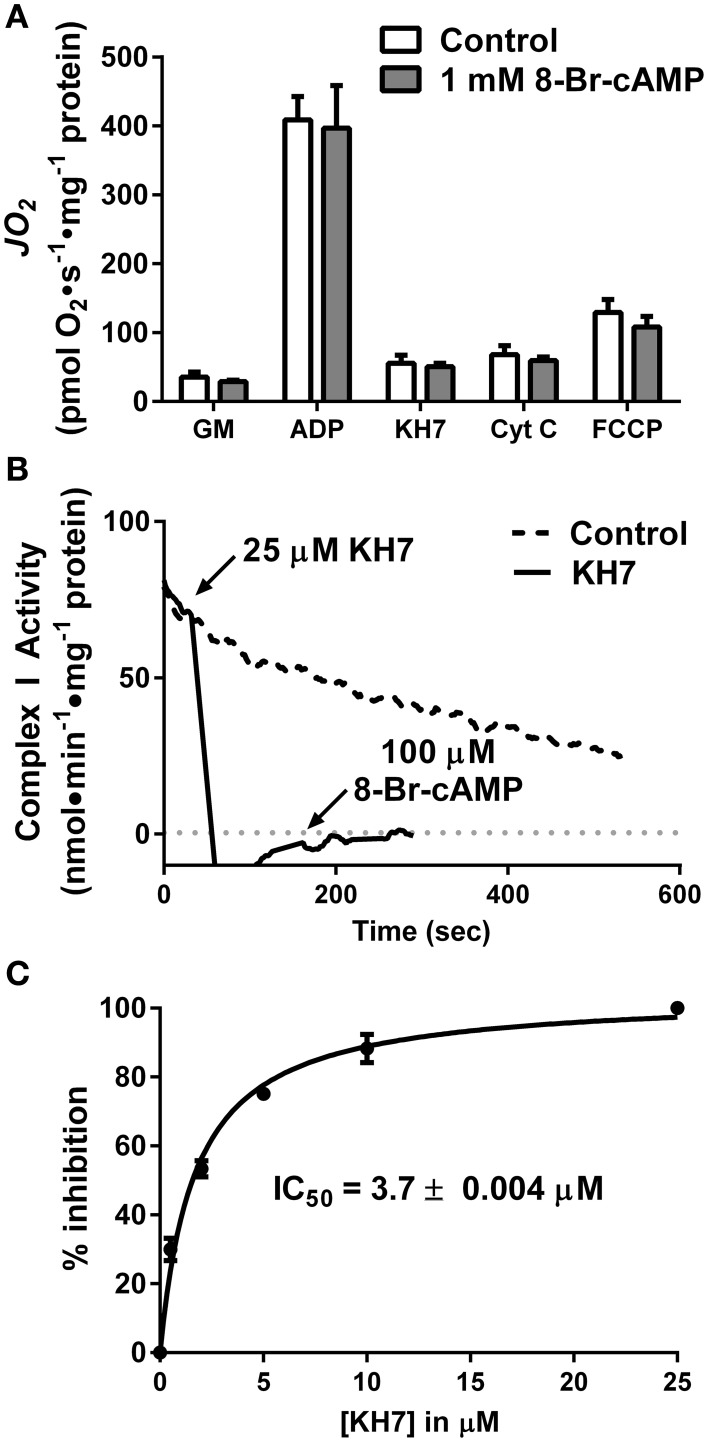
**The soluble adenylyl cyclase inhibitor KH7 inhibits complex I activity independent of cAMP/PKA signaling**. **(A)** *J*O_2_ was measured during a step-wise titration protocol in isolated liver mitochondria in the absence (white bars) or presence (gray bars) of 1 mM 8Br-cAMP. Each sequential addition was made after reaching a steady-state rate of O_2_ flux. *N* = 4/condition. **(B)** Complex I activity was determined by the oxidation rate of NADH in fragments of isolated skeletal muscle mitochondria following no treatment (dashed line) or sequential addition of 25 μM KH7 and 100 μM 8Br-cAMP (solid line). **(C)** Dose-response inhibition curve generated for inhibition of complex I activity as a function of KH7 concentration. *N* = 3 separate observations.

First, rates of oxygen consumption (*J*O_2_) were measured in isolated liver mitochondria in the absence or presence of 1 mM 8Br-cAMP, a membrane-permeable cAMP mimetic (Figure 2A). Surprisingly, 8Br-cAMP did not alter basal or maximal ADP-stimulated glutamate/malate-supported respiration. Similar to previous findings (Acin-Perez et al., 2009b), KH7 nearly completely inhibited ADP-stimulated respiration. Curiously however, the effect was not reversed or attenuated by the addition of 8Br-cAMP, which should bypass the inhibition of sAC. Addition of cytochrome c or FCCP also failed to restore respiration in the presence of KH7. Together, these findings suggest that the inhibitory effect of KH7 occurs independent of sAC/cAMP/PKA signaling and is not associated with loss of mitochondrial membrane integrity.

To further define the mechanism by which KH7 acts independently of cAMP signaling (Tian et al., 2011; Di Benedetto et al., 2013), complex I activity was measured in freeze-fractured fragments of isolated skeletal muscle mitochondria in the absence or presence of KH7. Addition of KH7 led to an immediate ablation of complex I activity that was not recovered by the addition of 8Br-cAMP (Figure 2B). Dose-response curves for complex I activity as a function of KH7 concentration in PKA-depleted mitochondrial fragments revealed an IC_50_ value of 3.7 μM (Figure 2C), comparable to previously reported IC_50_ values of KH7 for sAC (Hess et al., 2005; Bitterman et al., 2013). These findings suggest that the effects of KH7 on mitochondrial respiration are mediated by direct inhibition of complex I.

**Figure 3:**
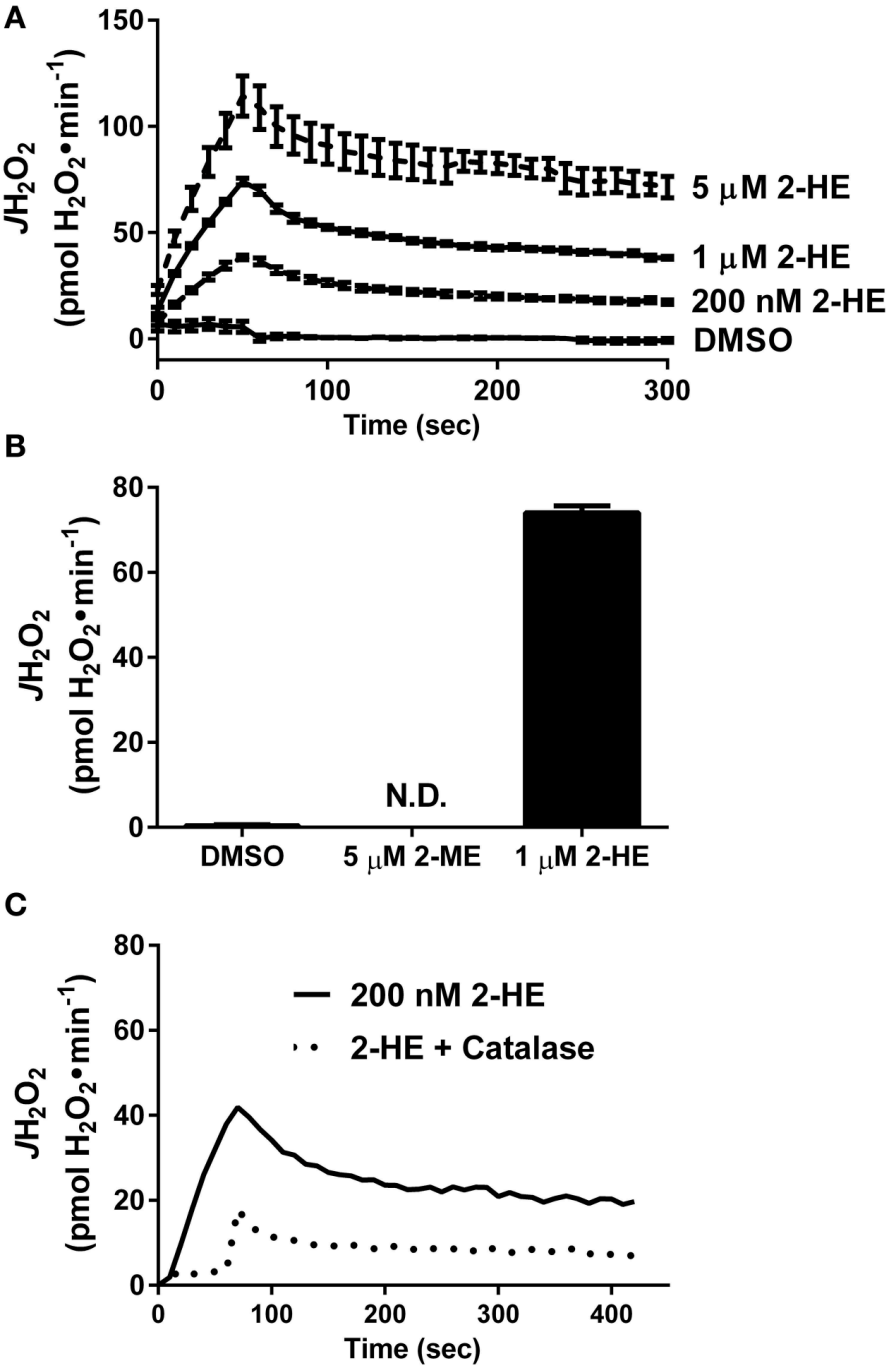
**The soluble adenylyl cyclase inhibitor 2-hydroxyestradiol (2-HE) produces H_2_O_2_ in a cell-free system**. **(A)** Comparison of H_2_O_2_ production from 2-methoxyestradiol (2-ME) and 2-hydroxyestradiol (2-HE). **(B)** Dose-dependent rate of H_2_O_2_ production of 2-HE over a 5-min span. Results in **(A,B)** are the product of *N*=3 separate experiments. **(C)** H_2_O_2_ production by 200 nM 2-HE in the absence (solid line) or presence (dashed line) of 100 U/ml catalase.

Besides KH7, the only other known sAC inhibitor with an IC_50_ below 10 μM is the naturally occurring estrogen metabolite 2-hydroxyestradiol (2-HE) (Steegborn et al., 2005). Anecdotal reports have suggested that 2-HE is also capable of generating oxidant species via redox cycling (Fussell et al., 2011), although this has not been demonstrated experimentally. Using a cell/organelle-free based H_2_O_2_ detection system, we found that 2-HE, but not its metabolite 2-methoxyestradiol (2-ME), generates H_2_O_2_ spontaneously (Figure 3A) in a dose-dependent (Figure 3B) and catalase-sensitive (Figure 3C) manner. H_2_O_2_ production was detected with as little as 200 nM 2-HE, a concentration more than 50-fold lower than has been previously used to inhibit sAC in cell-based assays (Tian et al., 2011; Di Benedetto et al., 2013). These findings raise significant concerns regarding the specificity and use of both KH7 and 2-HE as tools to study cAMP-related signaling events.

### Inhibition of PKA decreases complex I-supported respiratory capacity

**Figure 4:**
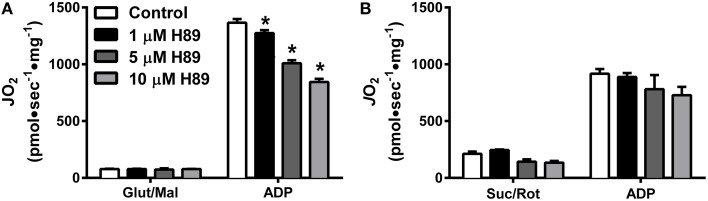
**PKA regulates Complex I- but not Complex II-supported respiration in isolated liver mitochondria**. *J*O_2_ was measured with complex I **(A)** or complex II **(B)** supported substrates in the absence (white bars) or presence of 1 (black bars), 5 (dark gray bars), or 10 (light gray bars) μM H89. *N* = 4–6/condition. * denotes *p* < 0.05 compared to control.

To further explore the potential impact of kinase activity on mitochondrial function, we focused our attention on PKA. Incubation of HeLa cells for 30 min with 1 μM H89, a PKA inhibitor (Chijiwa et al., 1990), has been shown to decrease mitochondrial respiratory capacity (Acin-Perez et al., 2009b), but the specific site(s) of regulation remains unknown. Using mitochondria isolated from liver, H89 dose-dependently decreased ADP-stimulated respiration supported by complex I (Figure 4A), but not complex II (Figure 4B) substrates. These findings prompted us to specifically focus on the role of PKA in the regulation of complex I activity.

### H89-mediated PKA inhibition alters ADP-supported respiratory kinetics

**Figure 5:**
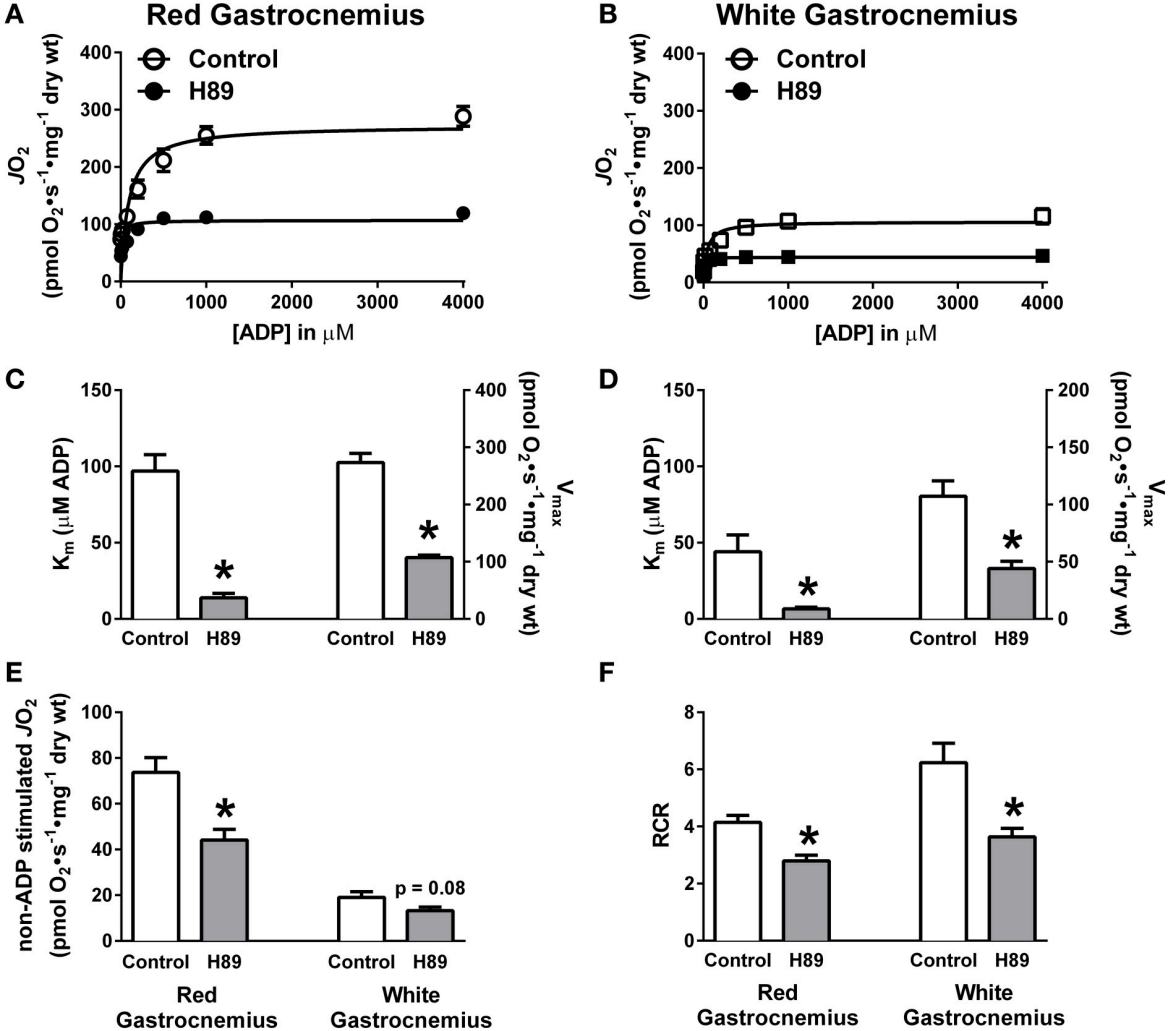
**PKA regulates ADP kinetics, proton leak and respiratory control ratio in oxidative and glycolytic mouse PmFBs**. ADP titration experiments were performed in RG **(A)** and WG **(B)** PmFBs with pyruvate and malate in the absence (open symbol) or presence (closed symbol) of 10 μM H89 in the assay media. Michaelis Menten-like kinetics generated from ADP titration experiments in RG **(C)** and WG **(D)**. **(E)** Non-ADP stimulated *J*O_2_ was compared from ADP titration experiments in the absence (white bars) or presence (gray bars) of 10 μM H89 in RG (left) and WG (right). **(F)** Respiratory control ratio (ADP-stimulated *J*O_2_/non-ADP stimulated *J*O_2_) was calculated from ADP titration experiments in the absence (white bars) or presence (gray bars) of 10 μM H89 in RG (left) and WG (right). *N* = 4–6/condition. * denotes *p* < 0.05 compared to Control.

To further define the impact of PKA inhibition on complex I-supported respiration, ADP titration experiments were performed on H89-treated PmFBs during respiration supported by pyruvate/malate. Both slow-twitch (RG) and fast-twitch (WG) PmFBs were used for these studies because the metabolic phenotype (e.g., oxidative vs. glycolytic) of the muscle governs both respiratory kinetics (Kuznetsov et al., 1996) and H_2_O_2_ emitting potential (Anderson and Neufer, 2006). Initial examinations revealed a decrease in respiration at and above an ADP concentration of 75 μM in RG (Figure 5A) and 200 μM in WG (Figure 5B). Applying Michaelis Menten-like kinetic analyses, these data were further dissected to yield maximal respiratory capacity (V_max_) and sensitivity to ADP (apparent K_m_—the ADP concentration required to elicit 50% of V_max_) (Kuznetsov et al., 1996). H89 treatment decreased both the apparent K_m_ and V_max_ in RG (Figure 5C) and WG (Figure 5D), indicating an increased sensitivity to ADP but decreased maximal respiratory capacity. H89 treatment decreased non-ADP stimulated respiration in RG with a similar, but non-significant (*p* = 0.08), trend in WG (Figure 5E). Finally, H89 treatment decreased respiratory control ratio (RCR), an index of mitochondrial coupling, in both RG and WG (Figure 5F). Altogether, these data suggest that inhibition of PKA decreases respiration supported by complex I, but not complex II, and does so during both proton leak- and ADP-dependent respiration.

### H89-mediated PKA inhibition alters complex I substrate kinetics

**Figure 6:**
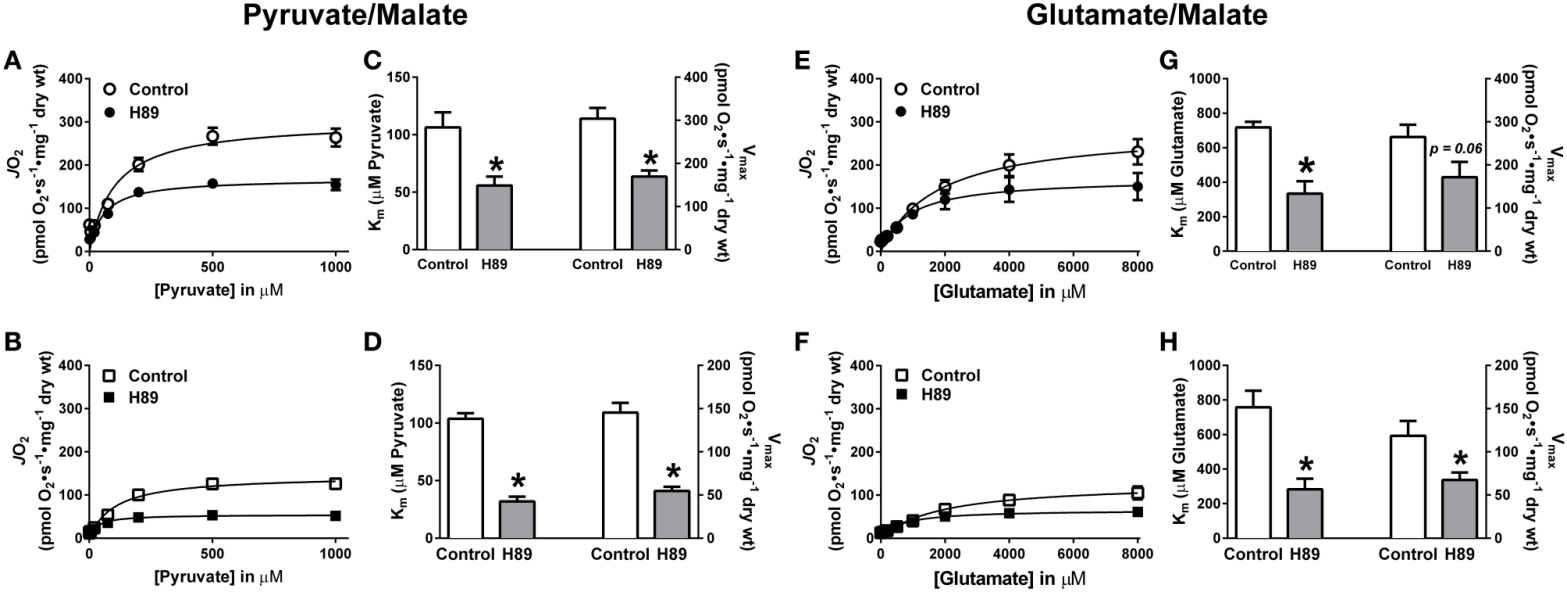
**Effects of PKA inhibition on substrate oxidation kinetics in mouse PmFBs**. Pyruvate titrations were performed in PmFBs from RG **(A)** and WG **(B)** in the absence (open symbol) or presence (closed symbol) of 10 μM H89 in the assay media. K_m_ and V_max_ were determined based on Michaelis-Menten like kinetics in RG **(C)** and WG **(D)**. *N* = 4–8/condition. ^*^ denotes *p* < 0.05 compared to Control. Glutamate titrations were performed in RG **(E)** and WG **(F)** in the absence (open symbol) or presence (closed symbol) of 10 μM H89. K_*m*_ and V_max_ were determined based on Michaelis-Menten like kinetics in RG **(G)** and WG **(H)**. *N* = 4–8/condition. * denotes *p* < 0.05 compared to Control.

To further explore the possibility that PKA regulates complex I, pyruvate and glutamate titrations were performed under ADP-stimulated conditions in RG and WG PmFBs in the absence or presence of H89. In both RG (Figure 6A) and WG (Figure 6B), inhibition of PKA decreased respiration at or above a pyruvate concentration of 100 μM. This was accompanied by an increase in sensitivity to pyruvate and a decrease in respiratory capacity in both tissues (Figures 6C,D). Glutamate titration experiments yielded similar data (Figures 6E–H). These findings using two distinct NADH-linked substrates provide evidence that PKA-mediated phosphorylation influences complex I-supported respiratory kinetics.

### H89-mediated PKA inhibition decreases H_2_O_2_ production during reverse electron flow

**Figure 7:**
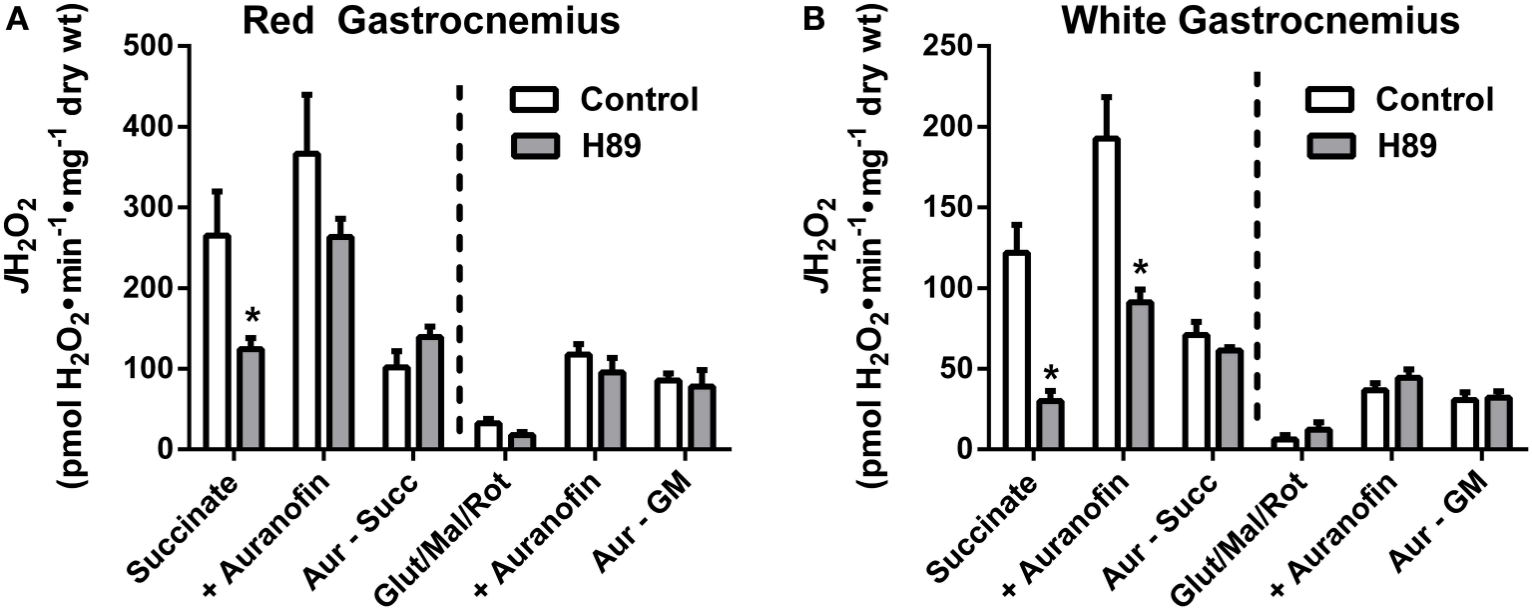
**PKA signaling regulates reverse, but not forward electron flow, through complex I in mouse PmFBs**. Mitochondrial H_2_O_2_ emitting potential (mOEP) was measured in RG **(A)** and WG **(B)** with succinate (left) or glutamate/malate/rotenone (right) and in the absence (white bars) or presence (gray bars) of 10 μM H89 in the assay media. Auranofin (1 μM) was added to specifically measure H_2_O_2_ production, and an “oxidant scavenging index” was determined based on the increase in mOEP following addition of auranofin. *N* = 4–8/condition. * denotes *p* < 0.05 compared to Control.

With evidence suggesting a role for PKA in the regulation of respiratory kinetics, particularly at complex I, we next sought to examine whether PKA affects the susceptibility of complex I to electron leak and H_2_O_2_ production/emission during reverse (i.e., succinate) or forward (glutamate/malate/rotenone) electron flow. In PmFBs from both RG (Figure 7A) and WG (Figure 7B), inhibition of PKA decreased H_2_O_2_ emission during reverse, but not forward, electron flow. Addition of auranofin to inhibit mitochondrial H_2_O_2_ scavenging elicited similar increases in H_2_O_2_ emission in the absence or presence of H89. Total mitochondrial oxidant scavenging was also not affected by H89. Together these data indicate that PKA inhibition affects H_2_O_2_ production, not scavenging, and that PKA may mediate its effects on mitochondrial energetics, at least in part, via regulation of electron flow at or near the interface between complex I and the Q-pool.

## Discussion

In recent years, starting with the discovery of sAC (Buck et al., 1999), a potential role for cAMP signaling in the mitochondrial matrix has emerged (Valsecchi et al., 2013). Several reports have described a role for sAC within mitochondria (Zippin et al., 2003; Acin-Perez et al., 2009b; Di Benedetto et al., 2013), the existence of mitochondrial cAMP/PKA signaling microenvironments (Papa et al., 1999; Livigni et al., 2006; Di Benedetto et al., 2008; Acin-Perez et al., 2011), and a wide variety of reversibly phosphorylated mitochondrial proteins (Zhao et al., 2011; Grimsrud et al., 2012). In contrast with plasma membrane-bound G-protein-linked forms of AC, sAC is activated by bicarbonate and calcium (Litvin et al., 2003). CO_2_ produced by the TCA cycle, and subsequent conversion to ${\text{HCO}}_{3}^{-}$ by carbonic anhydrase, has been suggested as a mechanism by which sAC/PKA signaling is activated in mitochondria (Acin-Perez et al., 2009b). In the present study however, evidence is provided that mitochondrial cAMP/PKA signaling is not activated by increased flux through the TCA cycle. In addition, two widely used inhibitors of sAC were found to have distinct non-specific effects that limit their utility in studies of mitochondrial function. Notably however, pharmacological inhibition of PKA was found to alter OXPHOS kinetics during respiration supported by NADH-linked substrates and H_2_O_2_ emission during reverse electron flow through complex I, providing additional evidence that complex I may be regulated by reversible phosphorylation.

The discovery and subsequent characterization of sAC within specific cellular organelles has led to the concept of compartmentalized cAMP signaling. A seminal finding in this field was that exogenous ${\text{HCO}}_{3}^{-}$ can increase cAMP levels via activation of sAC (Chen et al., 2000), a discovery that has been confirmed in multiple subsequent studies (Litvin et al., 2003; Zippin et al., 2003; Di Benedetto et al., 2013), including this report (Figure 1A). A central premise of the sAC-cAMP-PKA axis is that endogenous ${\text{HCO}}_{3}^{-}$ generated during increased flux through the TCA cycle is responsible for activating sAC. Here we directly tested this hypothesis and found that even during maximal uncoupled respiration, and in the presence of multiple substrate combinations that feed into the TCA cycle, mitochondrial cAMP levels did not change (Figure 1B). ATP was included in the assay at a concentration (1 mM) sufficient to provide substrate for sAC without inducing substrate inhibition (>5 mM) (Litvin et al., 2003). These findings therefore suggest that endogenous production of CO_2_ from the TCA cycle does not activate sAC in skeletal muscle mitochondria. More recent findings have provided evidence that an increase in the frequency and amplitude of matrix Ca^2+^ oscillations, as would occur during muscle contractions, is likely the more physiologically important regulator of sAC in mitochondria (Di Benedetto et al., 2013).

Defining the role of sAC in the regulation of mitochondrial bioenergetics has also hinged greatly on the use of two compounds marketed as sAC inhibitors: KH7 (Hess et al., 2005; Acin-Perez et al., 2009a,b, 2010) and 2-HE (Steegborn et al., 2005; Tian et al., 2011; Di Benedetto et al., 2013). In this report, we provide evidence demonstrating that both KH7 and 2-HE have distinct non-specific effects that preclude their use for studying mitochondrial energetics. First, KH7 directly inhibits mitochondrial respiration independent of cAMP/PKA signaling (Figure 2A) (Di Benedetto et al., 2013), and it appears to do so via direct inhibition of complex I (Figures 2B,C). Second, 2-HE, a naturally occurring estrogen metabolite, spontaneously generates high rates of H_2_O_2_ (Figures 3A–C), potentially affecting redox buffering systems and thus the oxidation state of mitochondrial proteins. Previous studies (Hess et al., 2005; Steegborn et al., 2005; Acin-Perez et al., 2009a,b, 2010; Tian et al., 2011; Di Benedetto et al., 2013) using one or both of these compounds to examine the link between cAMP/PKA signaling and mitochondrial function should therefore be interpreted with caution. The recently elucidated crystal structure of human sAC during catalysis and activation via ${\text{HCO}}_{3}^{-}$ (Kleinboelting et al., 2014) will hopefully facilitate the development of sAC inhibitors with greater specificity.

The impact of membrane permeable cAMP analogs on mitochondrial bioenergetics has also produced conflicting data. In both intact cells and isolated mitochondria, Acin-Perez et al. (2009b) found that 8Br-cAMP induced a slight but statistically significant increase in respiration under both basal and maximally-stimulated respiration conditions. ATP synthesis rate and mitochondrial membrane potential under non-phosphorylating conditions were also increased by 8Br-cAMP (Acin-Perez et al., 2009b). Di Benedetto et al. (2013) however failed to observe any impact of 8Br-cAMP, or several other more permeable analogs, on mitochondrial ATP concentration in intact cells. In the present study, 8Br-cAMP also failed to increase either basal or ADP-stimulated respiration in isolated liver mitochondria (Figure 2A).

However, the notion that a mitochondrial matrix sAC-cAMP-PKA axis regulates OXPHOS is supported by multiple lines of direct and indirect evidence (Raha et al., 2002; Acin-Perez et al., 2009b; Valenti et al., 2011; Di Benedetto et al., 2013), including the recent finding that numerous electron transport proteins in mouse liver and skeletal muscle originally identified in the MitoCarta (Pagliarini et al., 2008) have PKA consensus phosphorylation sites (Zhao et al., 2011; Grimsrud et al., 2012). Using cAMP-specific FRET sensors, two groups (Di Benedetto et al., 2013; Lefkimmiatis et al., 2013) have recently provided the most direct evidence that cAMP is produced inside mitochondria by sAC in response to increased matrix Ca^2+^ and, to a lesser extent, ${\text{HCO}}_{3}^{-}$. Additionally, literature spanning over 20 years implicates complex I as a target of PKA-dependent phosphorylation (Technikova-Dobrova et al., 1993; Sardanelli et al., 1995; Papa et al., 1999). In the current study, PKA inhibition was found to dose-dependently decrease complex I, but not complex II, supported respiration (Figures 4A,B), thus providing further direct evidence that PKA-mediated phosphorylation plays an important role in the regulation of complex I activity.

In PmFBs, H89-mediated inhibition of PKA elicited effects on mitochondrial respiratory kinetics, respiratory capacity, and oxidant emission that converged on complex I. Increased sensitivity of OXPHOS to both ADP (Figures 5C,D) and complex I-supported respiratory substrates (Figures 6C,D,G,H), combined with decreased maximal respiratory capacity, suggest a “bottleneck” in the ETS established by PKA inhibition. The effects of PKA inhibition were qualitatively similar in PmFBs from predominantly red oxidative and white glycolytic muscles, suggesting the mitochondrial phospho-regulatory mechanisms are similar in the two fiber types. To elucidate how PKA may regulate complex I-mediated electron flow, two substrate/inhibitor combinations were used to examine forward or reverse electron flow. The flavin (F) site of complex I is responsible for NADH reduction and the majority of electron leak from forward electron flow (Treberg et al., 2011). H_2_O_2_ emitting potential at this site was not affected by H89 (Figures 7A,B). The quinone (Q) site of complex I is responsible for donating electrons to the Q pool and accounts for the majority of electron leak that can occur from reverse electron flow during respiration supported by the complex II substrate succinate. Inhibition of PKA decreased electron leak from the Q-site during reverse electron flow but not from the F-site during forward electron flow (Figures 7A,B), suggesting that PKA regulates complex I activity somewhere between these two sites of electron transfer. This is of particular interest because the nuclear-encoded 18 kDa subunit of complex I physically lies in between these two sites of electron transfer, is exposed to the mitochondrial matrix (Baradaran et al., 2013), and is a physiologically relevant site of PKA-mediated phosphorylation (Sardanelli et al., 1995; Papa et al., 1999; Papa, 2002). Loss of the gene that encodes this subunit (NDUFS4) in mice replicates Leigh syndrome (Quintana et al., 2010; Johnson et al., 2013), a devastating human neurological, mitochondrial-linked disease. Although the NDUFS4 subunit is not thought to be directly involved in electron transfer, it is possible that PKA-mediated phosphorylation within this subunit alters electron transfer and/or (Lochner and Moolman, 2006) complex I function in a currently undefined manner.

The finding that PKA inhibition lowered mitochondrial *J*H_2_O_2_ emission in PmFBs is intriguing, as it suggests that activation of PKA signaling may accelerate mitochondrial *J*H_2_O_2_ emission. However, in the present studies, addition of 8Br-cAMP to activate PKA signaling failed to alter ADP-stimulated respiratory capacity, and accelerating TCA cycle flux failed to enhance cAMP levels. Along these same lines, calcium has recently been shown to enhance the driving forces of the oxidative phosphorylation system, although the effect is seen only when calcium is depleted from mitochodria prior to calcium stimulation (Glancy et al., 2013). Together, these findings were interpreted to suggest that calcium, and potentially PKA signaling, may be already relatively high/active in isolated mitochondria, and thus experiments to further activate PKA signaling were not pursued.

A caveat to experiments using H89 is that this drug is not entirely specific for PKA (Davies et al., 2000; Lochner and Moolman, 2006). However, the data provided are in agreement with previous reports demonstrating a role for PKA in the regulation of complex I in other tissues (Sardanelli et al., 1995; Papa et al., 1999; Papa, 2002). In the present study, H89 was chosen because a primary objective of this project was to compare findings in muscle PmFBs to previous findings in isolated liver mitochondria (Acin-Perez et al., 2009a,b; 2010; 2011). Interestingly, a recent report in isolated rat liver mitochondria suggests that hydrogen sulfide (H_2_S) is capable of regulating mitochondrial respiration (Módis et al., 2013), possibly via inhibition of phosphodiesterase 2A (PDE2A), a mitochondrial PDE isoform (Acin-Perez et al., 2011). Furthermore, in this report (Módis et al., 2013), the authors found that the inhibitory cAMP analog Rp-cAMP decreased complex II-supported respiration, although complex I-supported respiration was not reported. It remains to be seen whether PKA-mediated regulation of skeletal muscle complex I activity occurs with alternative PKA inhibitors like Rp-cAMP.

In conclusion, this report provides a novel collection of studies that: (1) challenge the notion that mitochondrial cAMP is regulated by TCA cycle flux, (2) reveal significant non-specific effects of widely used sAC inhibitors, and (3) provide the first functional evidence of PKA regulation of complex I in mouse muscle and liver mitochondria. These findings are of physiological significance particularly because they suggest that cAMP/PKA signaling not only regulates mitochondrial respiration, but also oxidant production. There is promise in the possibility that the mitochondrial cAMP/PKA axis can be manipulated to improve skeletal muscle metabolic health. Future studies await the development of targeted genetic approaches to more mechanistically examine the physiological role of mitochondrial cAMP-PKA signaling in health and disease.

## Author contributions

DL and DN designed the experiments with input from LR, TR, MT, CS, and CL. DL, LR, and TR performed experiments. DL, CL, and DN analyzed data and prepared figures. DL and DN drafted the manuscript. DL, TR, MT, CS, CL, and DN edited the paper to the final version.

## Funding

This research was supported by U.S. Public Health Services grant NIH R01 DK096907 (PDN).

### Conflict of interest statement

The authors declare that the research was conducted in the absence of any commercial or financial relationships that could be construed as a potential conflict of interest.
